# Supplementary material for: A brainstem to circadian system circuit links Tau pathology to sundowning-related disturbances in an Alzheimer’s disease mouse model
Source: Nat Commun. 2023 Aug 18;14:5027. doi: 10.1038/s41467-023-40546-w (PMC10439113; doi:10.1038/s41467-023-40546-w)
Supplement: Supplementary file 1 — Supplementary Information [file 41467_2023_40546_MOESM1_ESM.pdf]

Supplementary Figures and Tables

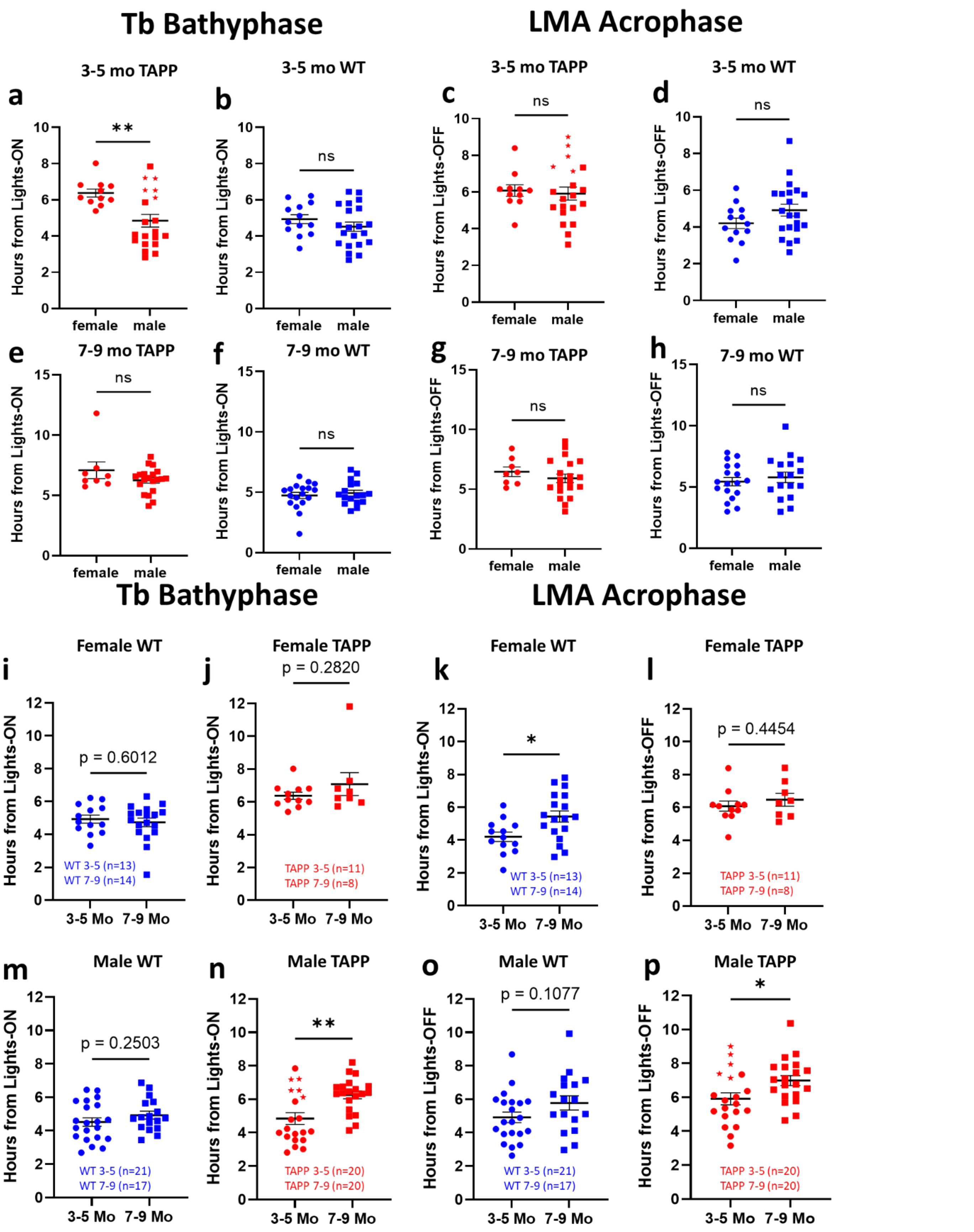

Figure S1. Effects of LPB pTau, aging, and sex on Tb bathyphase and LMA acrophase. a,b) 3-5mo TAPP females (red circles, n=8 mice) have later body temperature (Tb) bathyphases [Two-tailed unpaired t-tests, $t_{(29)}$=3.048, p=0.0049] than 3-5mo TAPP males (red squares/stars, n=20 mice). 3-5mo

wildtype (WT) females (blue circles, n=13 mice) and males (blue squares, n=21 mice) have similar [Two-tailed unpaired t-tests, $t_{(32)}$=1.106, p=0.2768]. **c,d)** 3-5mo TAPP females (red circles, n=8 mice) and males (red squares/stars, n=20 mice) [Two-tailed unpaired t-tests, $t_{(29)}$=0.3169, p=0.7536] and 3-5mo WT females (blue circles, n=13 mice) and males (red squares/stars, n=20 mice) [Two-tailed unpaired t-tests, $t_{(32)}$=1.528; p=0.1363] have similar locomotor activity (LMA) acrophases. **e,f)** 7-9mo TAPP females (red circles, n=8 mice) and males (red squares, n=20 mice) [Two-tailed unpaired t-tests, $t_{(26)}$=1.464, p=0.1552] and 7-9mo WT females (blue circles, n=18 mice) and males (blue squares, n=17 mice) [Two-tailed unpaired t-tests, $t_{(33)}$=0.5434, p=0.5905] have similar Tb bathyphases. **g,h)** 7-9mo TAPP females (red circles, n=8 mice) and males (red squares, n=20 mice) [Two-tailed unpaired t-tests, $t_{(26)}$=0.9012, p=0.3758], and 7-9mo WT females (blue circles, n=18 mice) and males (blue squares, n=17 mice) [Two-tailed unpaired t-tests, $t_{(33)}$=0.6345, p=0.5301] have similar LMA acrophases. **I,j.** Neither WT [Two-tailed unpaired t-tests, $t_{(29)}$=0.5285] nor TAPP [Two-tailed unpaired t-tests, $t_{(17)}$=0.1111] females develop later Tb bathyphases with aging. **k,l)** WT [Two-tailed unpaired t-tests, $t_{(29)}$=2.620, *p=0.0138] but not TAPP females [Two-tailed unpaired t-tests, $t_{(17)}$=0.7813] develop delayed LMA acrophases with aging. **m,n)** TAPP [Two-tailed unpaired t-tests, $t_{(38)}$=3.349, **p=0.0018] but not WT [Two-tailed unpaired t-tests, $t_{(36)}$=1.169] males develop delayed Tb bathyphases with consistent lateral parabrachial (LPB) hyperphosphorylated Tau (pTau). Red stars in (**n**) denote 3-5mo TAPP males with LPB pTau. **o,p)** TAPP [Two-tailed unpaired t-tests, $t_{(38)}$=2.304, *p=0.0268] but not WT [Two-tailed unpaired t-tests, $t_{(36)}$=1.650] males develop later LMA acrophases with consistent LPB pTau. Aging does not delay Tb nor LMA in TAPP females which have consistent LPB pTau at both ages **(Fig 2e,f,g,h)**. TAPP males develop delays with consistent LPB pTau **(Fig 2a,b,c,d**) and age. 3-5mo TAPP males with LPB pTau (red stars in **a**,**c**,**n**,**p**) have later Tb bathyphases and LMA acrophases compared to 3-5mo TAPP males without LPB pTau.

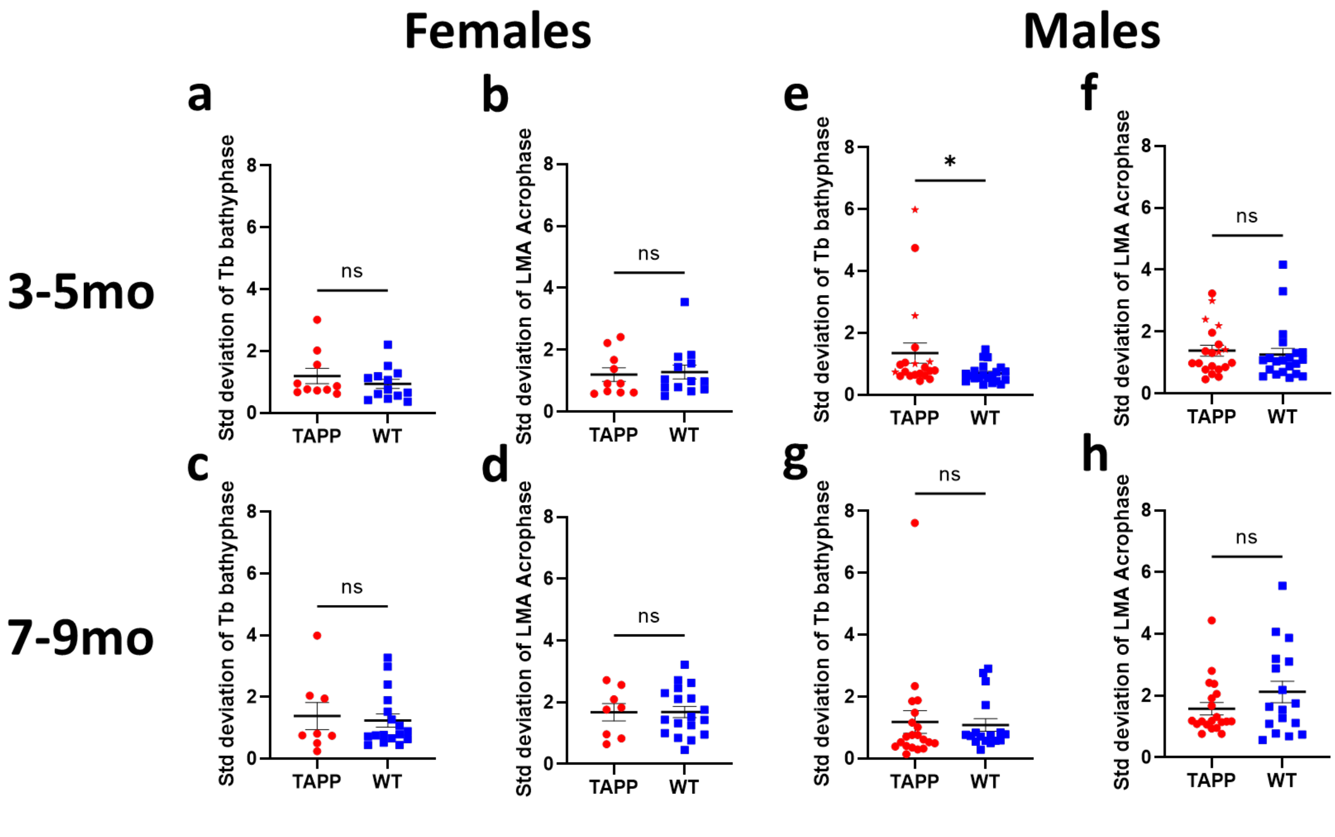

__Figure S2__. **Comparing the stability of Tb bathyphase and LMA acrophase measurements with standard deviations. a,b)** Standard deviations across the 7d recordings were not different between 3-

5mo TAPP females (red, n=10 mice) and 3-5mo wildtype (WT) females (blue, n=13 mice) for either body temperature (Tb) bathyphase [Two-tailed Mann-Whitney U-test, U=49, p=0.3434] or locomotor activity (LMA) acrophase [Two-tailed Mann-Whitney U-test, U=56; p=0.6049]. **c,d)** Standard deviations were also not different between 7-9mo TAPP females (red, n=8 mice) and 7-9mo WT females (blue, n=18 mice) for either Tb bathyphase [Two-tailed unpaired t-tests, $t_{(23)}$=0.3316, p=0.7432] or LMA acrophase [Two-tailed unpaired t-tests, $t_{(24)}$=0.02248, p=0.9823]. **e,f)** Standard Deviations of Tb bathyphase in 3-5mo TAPP males (red, n=20 mice) were greater than in 3-5mo WT males (blue, n=21 mice) [Two-tailed Mann-Whitney U-test, U=122, p=0.0213], but there were no differences for LMA acrophase [Two-tailed Mann-Whitney U-test, U=181, p=0.4614]. **g,h)** There were no differences in standard deviations between 7-9mo TAPP males (red, n=20 mice) and 7-9mo WT males (blue, n=17 mice) for either Tb bathyphase [Two-tailed Mann-Whitney U-test, U=138, p=0.3413] or LMA acrophase [Two-tailed Mann-Whitney U-test, U=143, p=0.4239].

**Groups**	3-5mo TAPP males (n = 20 mice)	3-5mo WT males (n = 21 mice)	3-5mo TAPP females (n = 11 mice)	3-5mo WT females (n = 13 mice)	7-9mo TAPP males (n = 20 mice)	7-9mo WT males (n = 16 mice)	7-9mo TAPP females (n = 8 mice)	7-9mo WT females (n = 18 mice)
**Tb acrophase (hours from LIGHTS-OFF)**	4.85 ± 0.35	4.52 ± 0.25	6.38 ± 0.22	4.93 ± 0.25	6.26 ± 0.23	4.97 ± 0.24	7.09 ± 0.70	4.74 ± 0.29
**LMA bathyphase (hours from LIGHTS-ON)**	5.91 ± 0.35	4.91 ± 0.32	6.08 ± 0.31	4.21 ± 0.29	6.98 ± 0.30	5.72 ± 0.41	6.47 ± 0.40	5.44 ± 0.39

Table S1. Additional phase markers of circadian entrainment, Tb acrophase and LMA bathyphase, show similar age and sex differences in TAPP and WT mice as Tb bathyphase and LMA acrophase. 3-5mo TAPP males did not show Tb acrophases that were significantly different from WT males at this age [Two-tailed unpaired t-test, $t_{(39)}$=0.7798, p=0.4402]. 3-5mo TAPP males did show significantly later LMA bathyphases than WT males [Two-tailed unpaired t test, $t_{(29)}$=2.095, p=0.0427], but like LMA acrophase, this appears to be driven by the subset of 5 males that already showed LPB pTau at this age (see Figure 4). 3-5mo TAPP females showed significantly later Tb acrophases (Two-tailed unpaired t test, $t_{(22)}$=4.295, p=0.0003) and LMA bathyphases (Two-tailed unpaired t test, $t_{(22)}$=4.448, p=0.0002) compared to WT females at this age. At 7-9mo, TAPP males also showed significantly later Tb acrophases (Two-tailed, unpaired t test, $t_{(35)}$=2.534, p=0.0005) and later LMA bathyphases (Two-tailed unpaired t test, $t_{(39)}$=0.7798, p=0.0159) compared to WT males at this age. 7-9mo TAPP females showed significantly later Tb bathyphases compared to 7-9mo WT females (Two-tailed unpaired t test, $t_{(34)}$=3.921, p=0.0006), but only showed a trend toward later LMA bathyphases (Two-tailed unpaired t test, $t_{(24)}$=1.792, p=0.0858). This may be due to later LMA bathyphases in WT females with age.

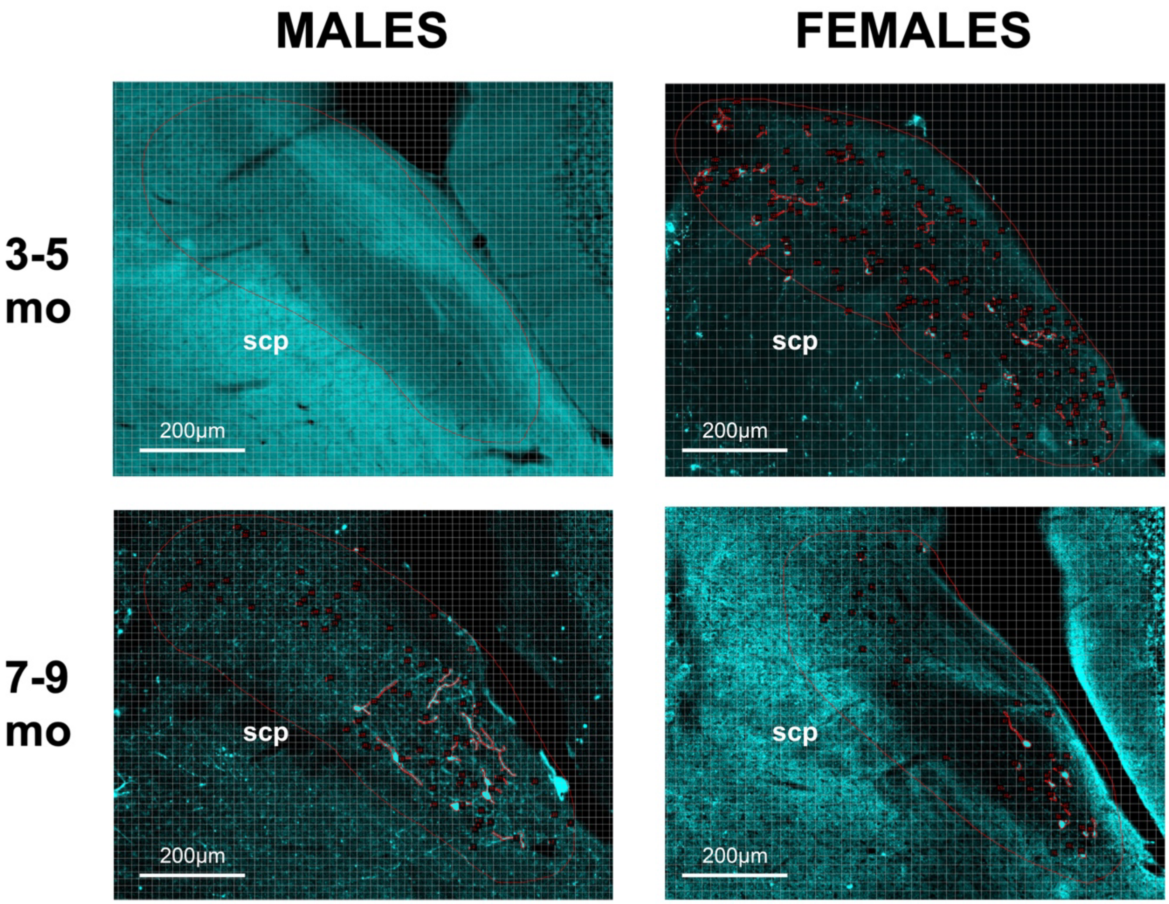

<u>Figure S3.</u> Representative examples of pTau quantification in the LPB. Same images depicted in **Figure 2a**. Following immunohistochemistry for hyperphosphorylated Tau (pTau, with the AT8 antibody, turquoise) images of the lateral parabrachial (LPB) were imported into SketchandCalc area quantification software. The boundary of the LPB was outlined (red) to delineate the quantification region and then cell bodies, axons, and dendrites expressing AT8 labelling were outlined in order to measure the total area within the LPB covered with pTau. n=15 3-5mo male mice, n=16 7-9mo male mice, n=6 3-5mo females mice, and n=4 7-9mo female mice.

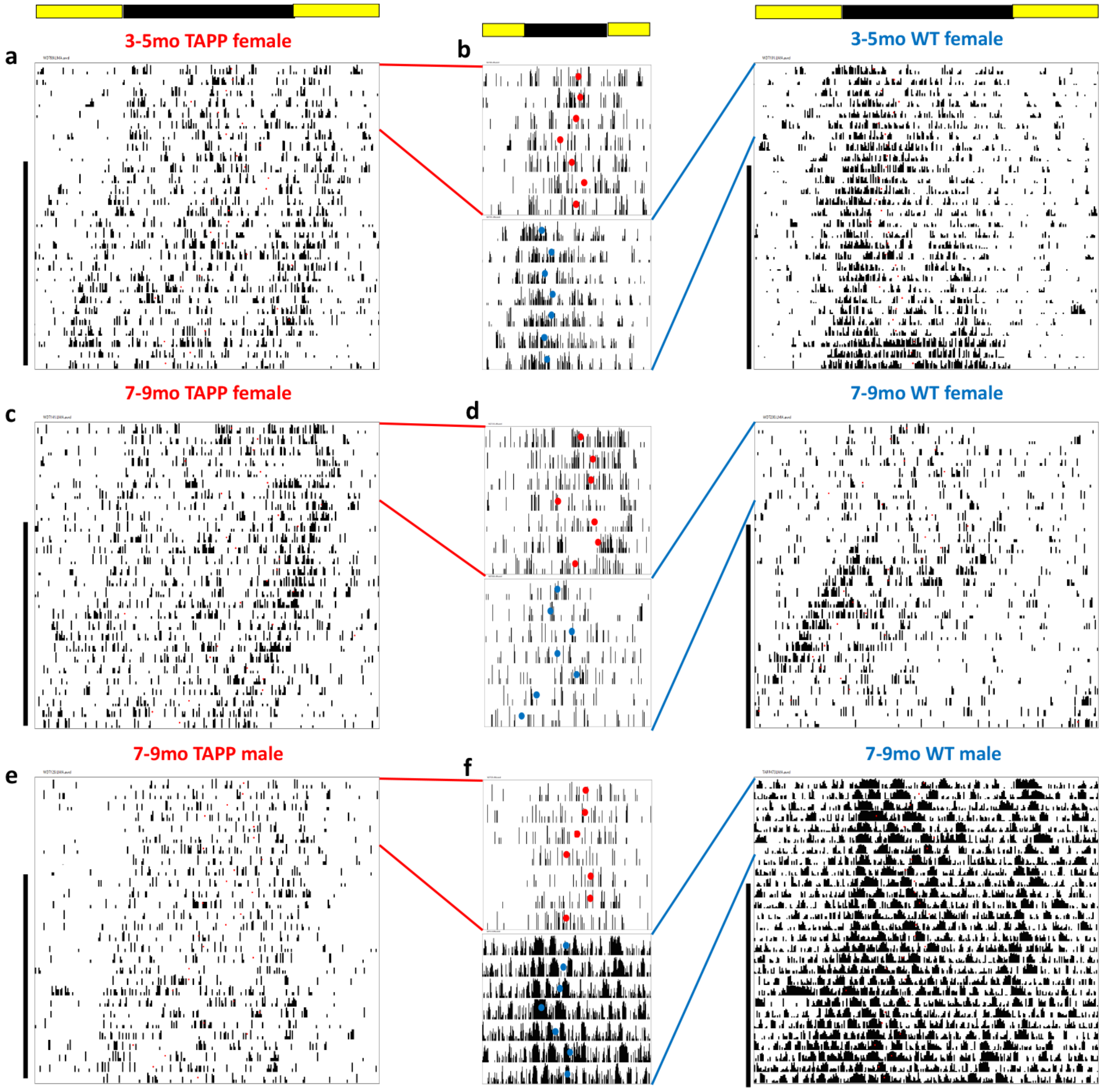

Figure S4. Representative LMA actograms of 3-5mo and 7-9mo female TAPP vs WT mice and 7-9mo TAPP vs WT mice, LMA acrophase denoted by red or blue dots respectively. a) Locomotor activity (LMA) recordings under 12h-12h light-dark (LD) and constant darkness (DD, denoted by black vertical bar) in a 3-5mo TAPP female (left red, n=11 mice) and 3-5mo wildtype (WT) female (right blue, n=13 mice). **b)** 7 days of LD recording for a 3-5mo TAPP female (top, red) and 3-5mo WT female (bottom blue). **c)** LD and DD recording of 7-9mo TAPP female (left red, n=8 mice) and 7-9mo WT female (right blue, n=18 mice). **d)** 7 days of LD recording for a 7-9mo TAPP female (top, red) and 7-9mo WT female (bottom blue). **e)** LD and DD recording of 7-9mo TAPP male (left red, n=20 mice) and 7-9mo WT male (right blue, n=17 mice). **f)** 7 days of LD recording for a 7-9mo TAPP male (top, red) and 7-9mo WT male (bottom blue).

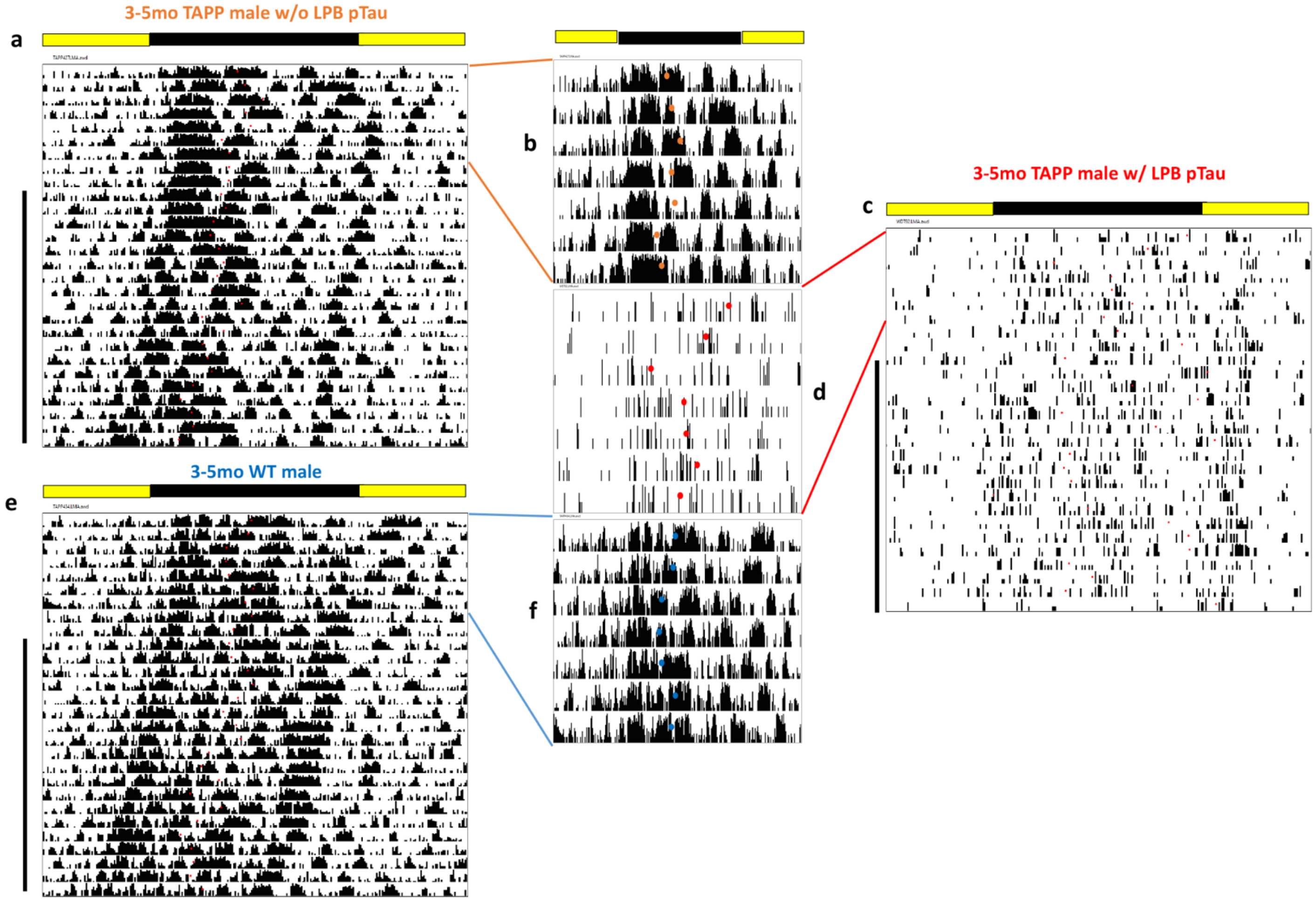

Figure S5. Representative LMA Actograms of 3-5mo TAPP male without LPB pTau, with LPB pTau and WT control with LMA acrophase denoted by orange, red, or blue dots respectively. a) Locomotor activity (LMA) recordings under 12h-12h light-dark (LD) and constant darkness (DD, denoted by black vertical bar) in a 3-5mo TAPP male without lateral parabrachial (LPB) hyperphosphorylated Tau (pTau) (n=15 mice). **b)** 7 days of LD recording for mouse in (a). **c)** LD and DD recording of LMA for a 3-5mo TAPP male with LPB pTau (n=5 mice) **d)** 7 days of LD recording for mouse in (c). **e)** LD and DD recording of LMA for a 3-5mo wildtype (WT) male (n=21 mice). **f)** 7 days of LD recording for mouse in (e).

**Groups**	3-5mo TAPP males (n = 20 mice)	3-5mo WT males (n = 21 mice)	3-5mo TAPP females (n = 10 mice)	3-5mo WT females (n = 13 mice)	7-9mo TAPP males (n = 20) mice	7-9mo WT males (n = 16 mice)	7-9mo TAPP females (n = 8 mice)	7-9mo WT females (n = 18 mice)
**Tb period length (hours)**	23.67 ± 0.04	23.75 ± 0.05	23.74 ± 0.04	23.66 ± 0.07	23.85 ± 0.03	23.80 ± 0.06	23.82 ± 0.09	23.69 ± 0.04
**LMA period length (hours)**	23.62 ± 0.04	23.74 ± 0.05	23.72 ± 0.06	23.68 ±0.06	23.84 ± 0.07	23.77 ± 0.10	23.88 ± 0.12	23.69 ± 0.07

<u>Table S2</u>. **Male and female TAPP and WT mice do not show significant differences in free-running period length of Tb or LMA at 3-5mo or 7-9mo.** After mice had been housed in constant darkness (DD) for at least 2 full weeks, a Chi-squared periodogram was performed on body temperature

(Tb) and locomotor activity (LMA) data over the next 10 consecutive days in DD. We detected no significant differences of free-running period length in 3-5mo males (Tb: Two-tailed unpaired t test, $t_{(39)}$=1.295, p=0.2029; LMA: Two-tailed unpaired t test, $t_{(39)}$=1.943, p=0.0593), 3-5mo females (Tb: Two-tailed unpaired t test, $t_{(21)}$=0.921, p=0.3675; LMA: Two-tailed unpaired t test, $t_{(21)}$=0.4214, p=0.6777), 7-9mo males (Tb: Two-tailed unpaired t test, $t_{(34)}$=0.772, p=0.2227; LMA: Two-tailed unpaired t test, $t_{(34)}$=0.6075, p=0.5475), or 7-9mo females (Tb: Two-tailed unpaired t test, $t_{(24)}$=0.1.872, p=0.0734; LMA: Two-tailed unpaired t test, $t_{(24)}$=1.511, p=0.1437).

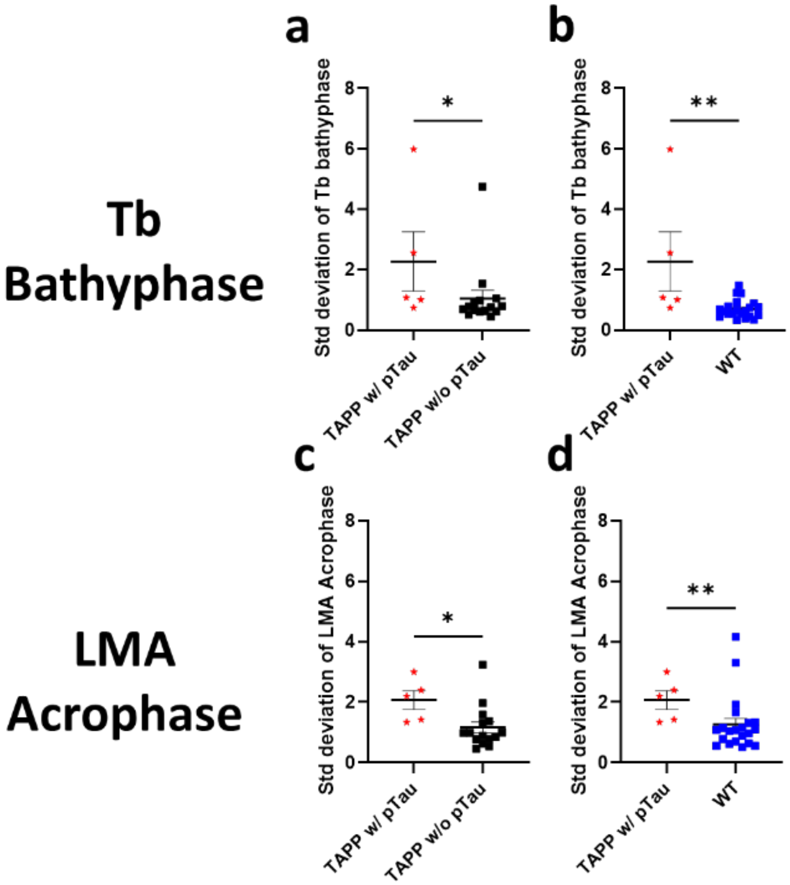

<u>Figure S6</u>. Phase markers are less stable in young male TAPP mice when pTau accumulation is at an early stage in the LPB. a,b) Across the 7d recordings under 12h-12h light-dark (LD), standard deviations of Tb bathyphase were elevated in 3-5mo TAPP males with lateral parabrachial (LPB) hyperphosphorylated Tau (pTau) (red stars, n=5 mice) compared to 3-5mo TAPP males without LPB pTau (black squares, n=15 mice) [Two-tailed Mann-Whitney U-test, U=14, *p=0.0418] and 3-5mo wildtype (WT) males (blue, n=21 mice) [Two-tailed Mann-Whitney U-test, U=14, **P=0.0099]. **c,d)** Standard deviations of LMA acrophase during the LD recordings were also elevated in 3-5mo TAPP males with LPB pTau (red stars, n=5 mice) compared to 3-5mo TAPP males without LPB pTau (black squares, n=15 mice) [Two-tailed Mann-Whitney U-test, U=10, *p=0.0146] and 3-5mo WT males (blue, n=21 mice) [Two-tailed Mann-Whitney U-test, U=14, **p=0.0099].

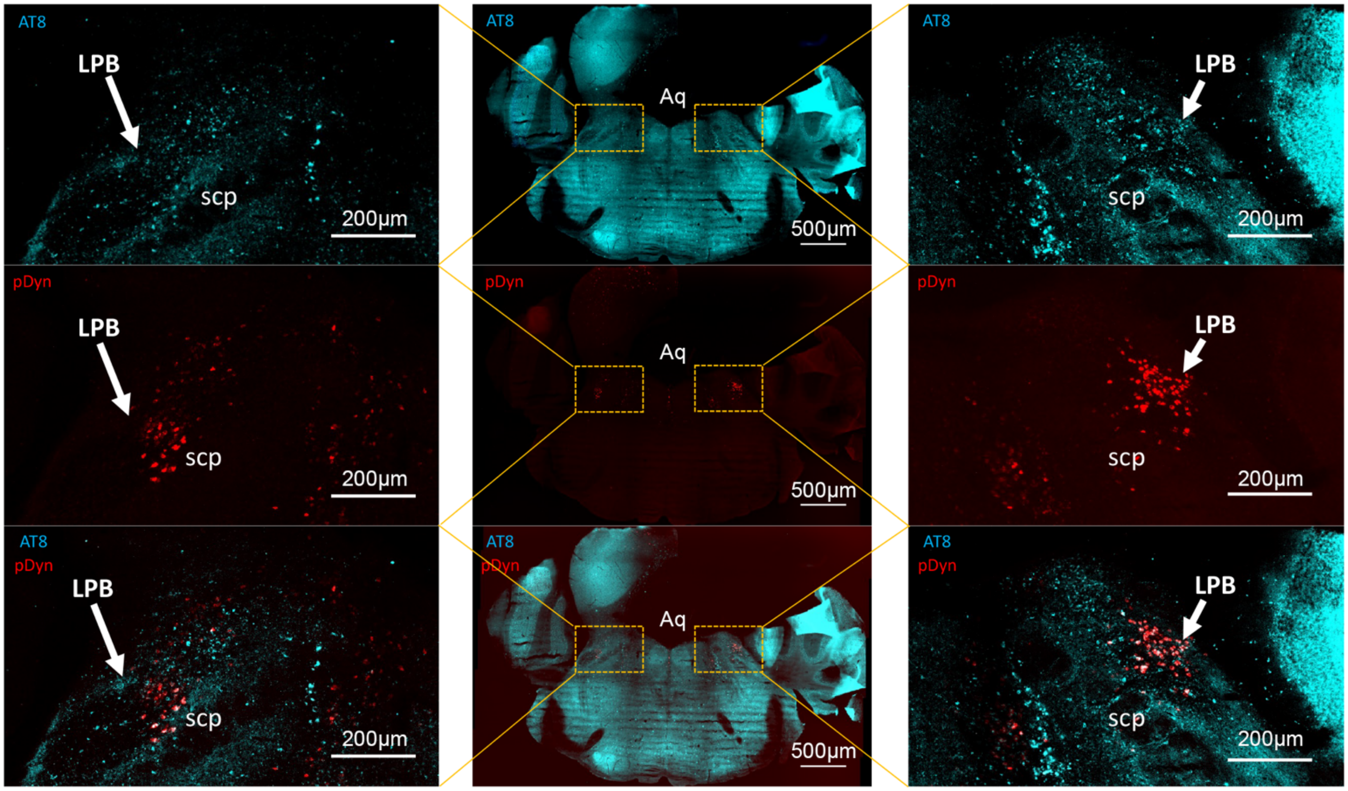

Figure S7. **Bilateral pTau colocalization with LPB^{dyn} cells in a 7–9mo male TAPP mouse**. (**Top row**) Hyperphosphorylated Tau (pTau) immunohistochemical staining (turquoise=AT8 antibody) shows numerous pTau-filled neurons in the bilateral lateral parabrachial (LPB, marked by white arrow). (**Middle row**) Prodynorphin (*pDyn*, red) RNAscope in situ hybridization shows anatomical location of *pDyn* neurons in the bilateral LPB (marked by white arrow). (**Bottom row**) Colocalization of pTau and *pDyn* neurons in the bilateral LPB (marked by white arrow). This is the same mouse shown in **Fig 5a**. scp: superior cerebellar peduncle. n=4 mice.

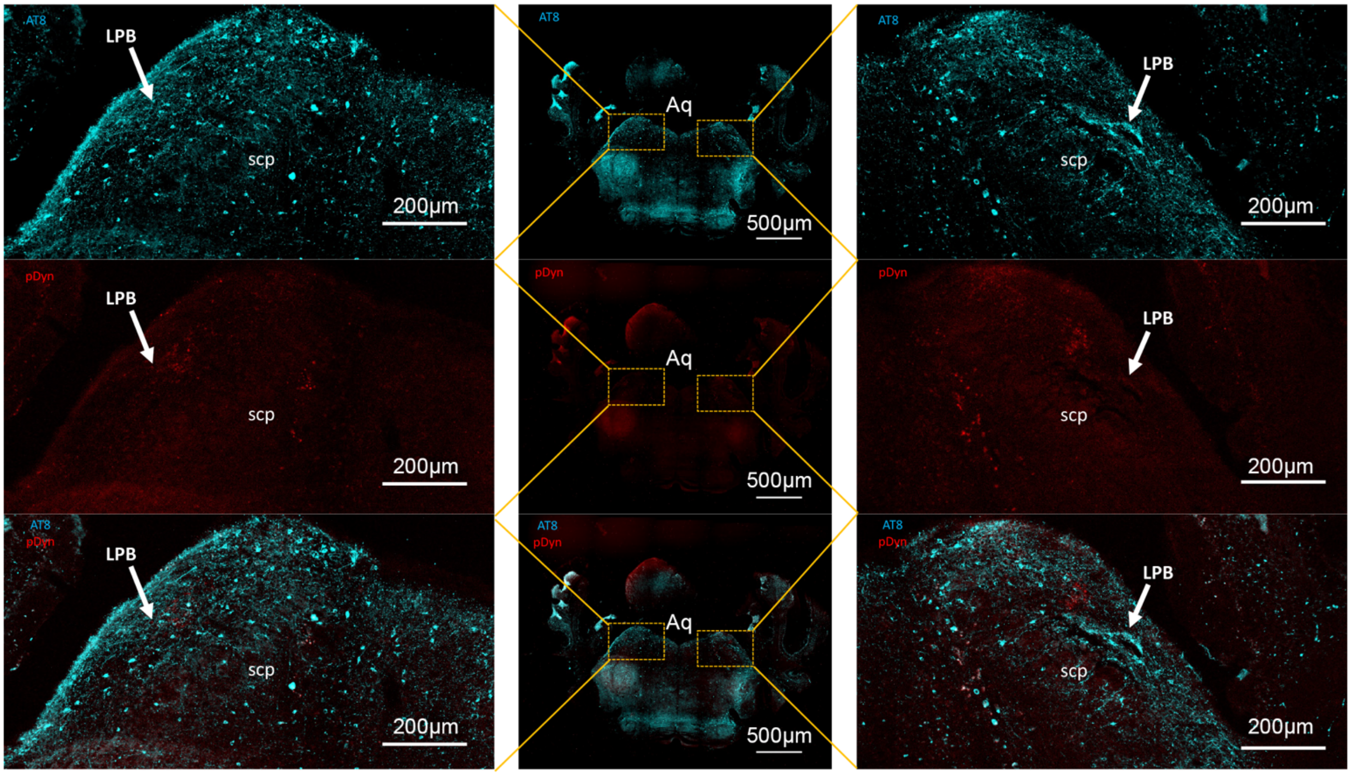

Figure S8. Bilateral pTau and pDyn staining in the LPB of a 13mo male TAPP mouse. (**Top row**) Hyperphosphorylated Tau (pTau) immunohistochemical staining (turquoise=AT8 antibody) shows numerous pTau-filled neurons in the bilateral LPB (marked with white arrow). (**Middle row**). Prodynorphin (pDyn, red) RNAscope in situ hybridization shows anatomical location of pDyn neurons in the bilateral LPB (marked by white arrow). Notice the stark reduction in labelling from LPB dynorphin (LPB^{dyn}) neurons corresponding to the neurodegeneration of these LPB^{dyn} cells. (**Bottom row**) Colocalization of pTau and pDyn neurons in the bilateral LPB (marked by white arrow). Compare this to **Supp Fig 2 (Bottom row)** to see time course of pTau filling numerous LPB^{dyn} neurons to the degeneration of these LPB^{dyn} neurons here. This is the same mouse shown in **Fig 5d**. scp: superior cerebellar peduncle. n=2 mice.

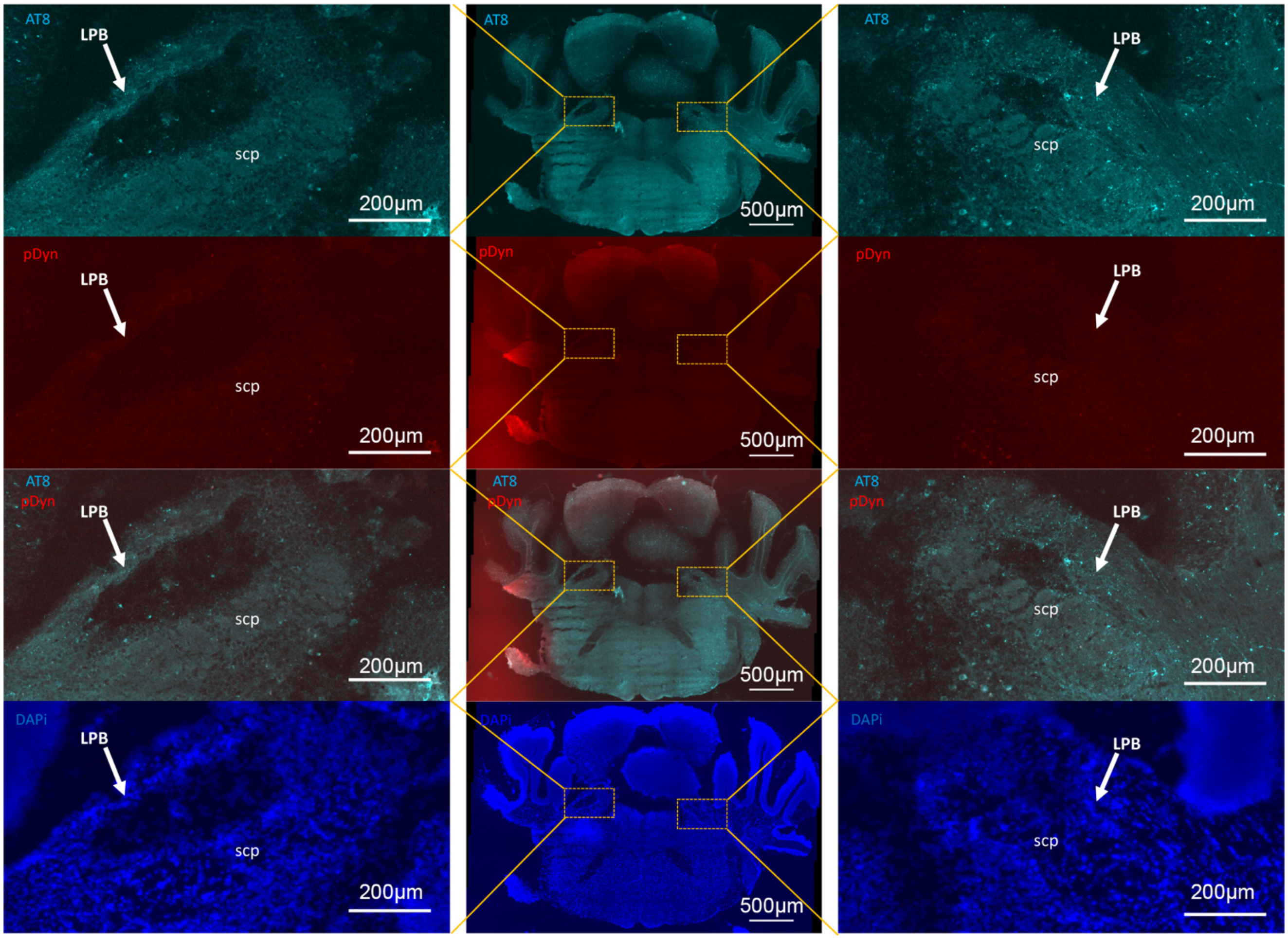

Figure S9. **Bilateral pTau and *pDyn* staining in the LPB of a 13mo female TAPP mouse**. (**First row**) Hyperphosphorylated Tau (pTau) immunohistochemical staining (turquoise=AT8 antibody) shows numerous pTau-filled neurons in the bilateral LPB (marked with white arrow). (**Second row**) Prodynorphin (*pDyn*, red) RNAscope in situ hybridization shows anatomical location of *pDyn* neurons in the bilateral LPB (marked by white arrow). Notice the stark reduction in labelling from LPB dynorphin (LPB^{dyn}) neurons corresponding to neurodegeneration of these LPB^{dyn} cells. (**Third row**) Colocalization of pTau and *pDyn* neurons in the bilateral LPB (marked by white arrow). Compare this to **Supp Fig 3 (Bottom row)** to see that this neurodegeneration of LPB^{dyn} neurons happens in both sexes. (**d**) DAPi staining (blue, labeling nuclei) to show striking neurodegeneration in the bilateral LPB. Notice how there is a clear bilateral lesion in the LPB region in each of the three channels shown (**First row, Second row, Fourth row**). Compare this to **Supp Fig 3 (Top and Middle rows)** to see that neurodegeneration appears to have affected the 13mo female more drastically. This is likely due to cell death beginning earlier in TAPP females compared to TAPP males. This also follows the same pattern of earlier phase delays and the earlier appearance of pTau in the LPB in TAPP females compared to TAPP males. This is the same mouse shown in **Fig 5e**. scp: superior cerebellar peduncle. n=2 mice.

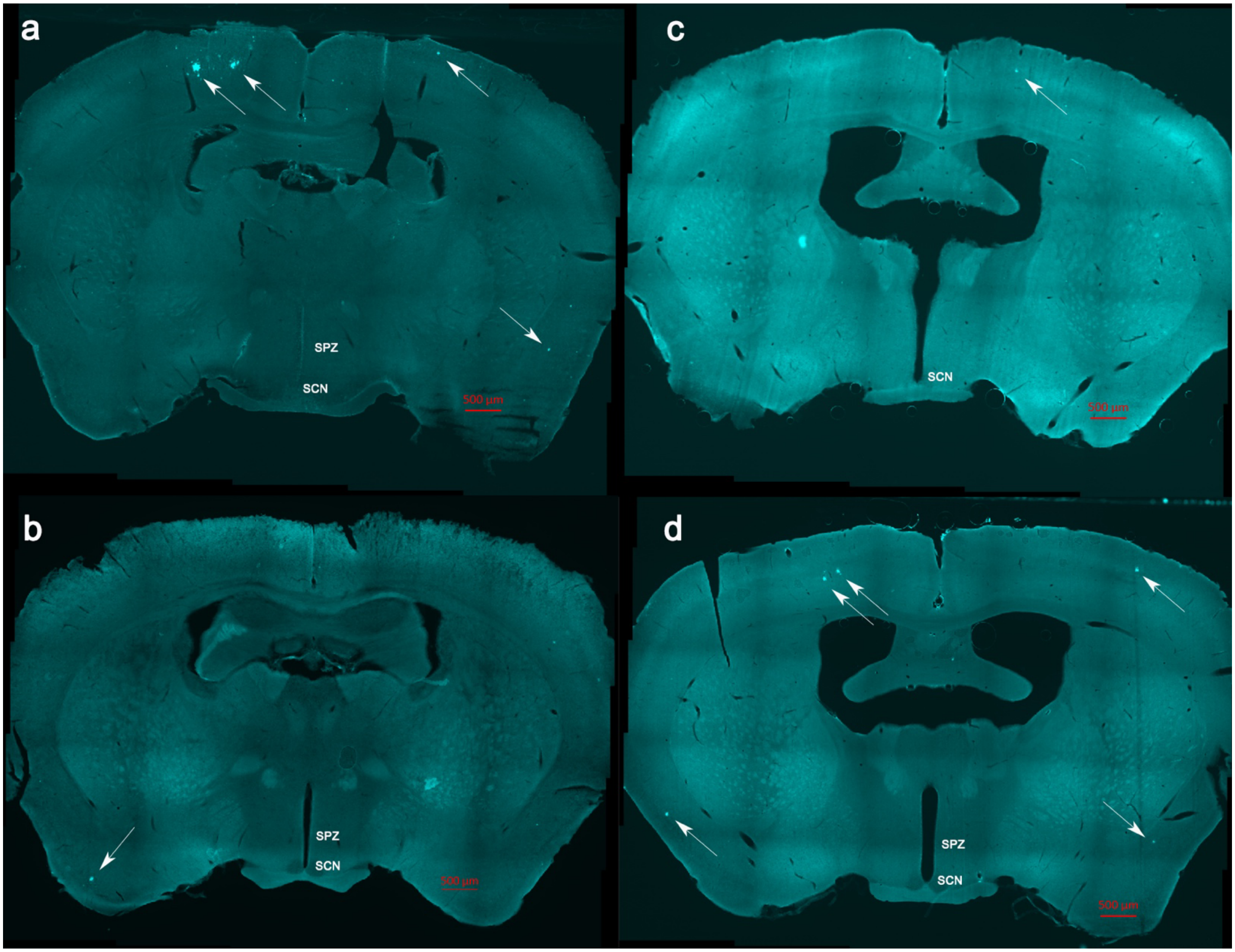

Figure S10. TAPP mice show similar sex differences in the development of Aβ pathology in cortical areas. a,b. 7-9mo male TAPP mice first show amyloid-beta (Aβ) pathology (turquoise=6E10 antibody) in cortical areas but not consistently in the same regions (white arrows). n=6 mice. **c.** Aβ pathology was detected in cortical areas in 3-5mo female TAPP mice (white arrow, n=4 mice), as well as in 7-9mo TAPP females (n=4 mice) (**d**), but not always in the same cortical areas in every mouse. Scale bar (red), 500µm.

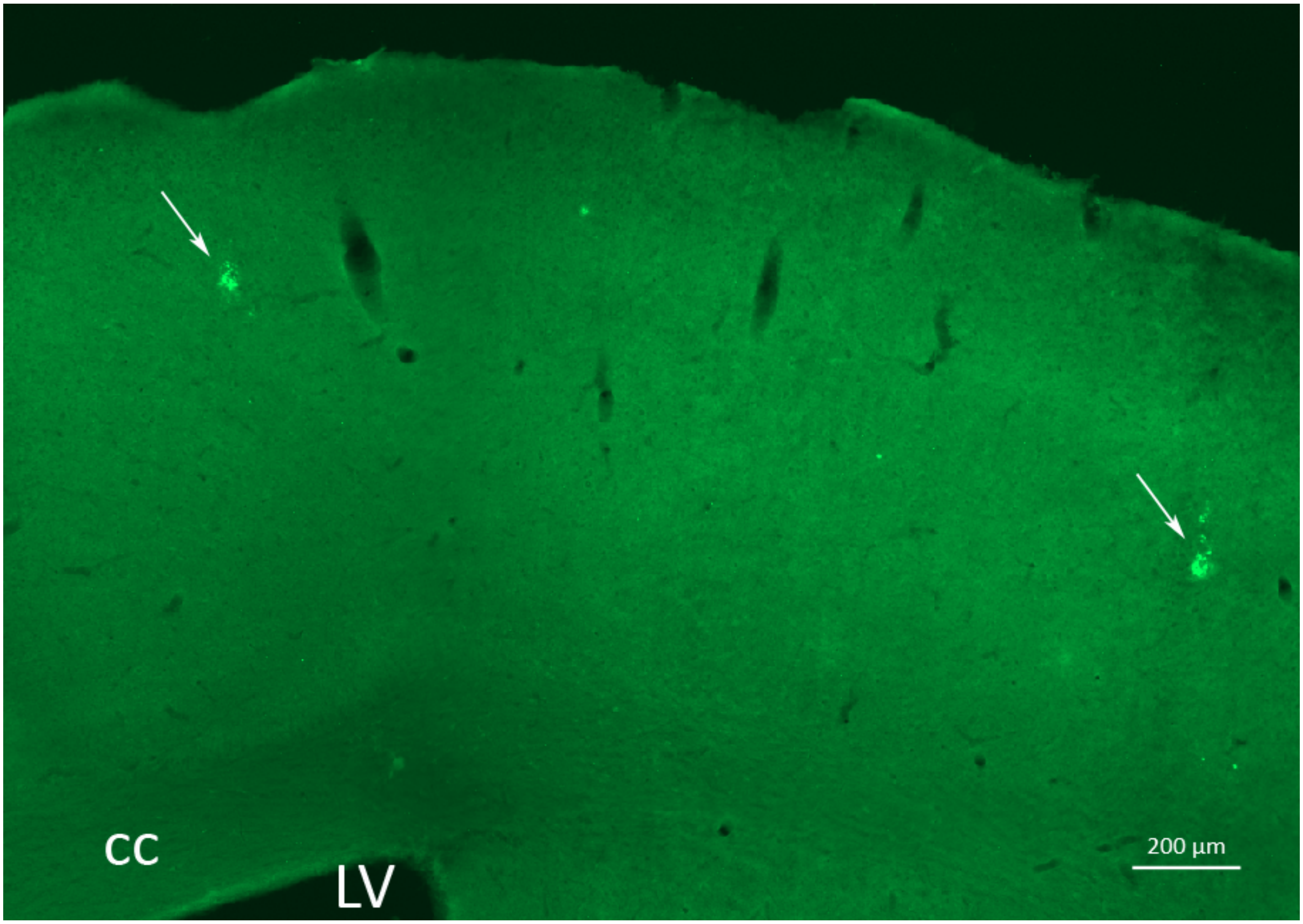

Figure S11. Aβ labeling in the cerebral cortex of TAPP mice with pTau in the LPB. Representative image from a 3-5mo TAPP female of amyloid-beta (Aβ) labeling with the 6E10 antibody showing Aβ plaques (white arrows) in the motor (left) and sensory (right) cortical regions. cc, corpus callosum. LV, lateral ventricle. n=4 mice.

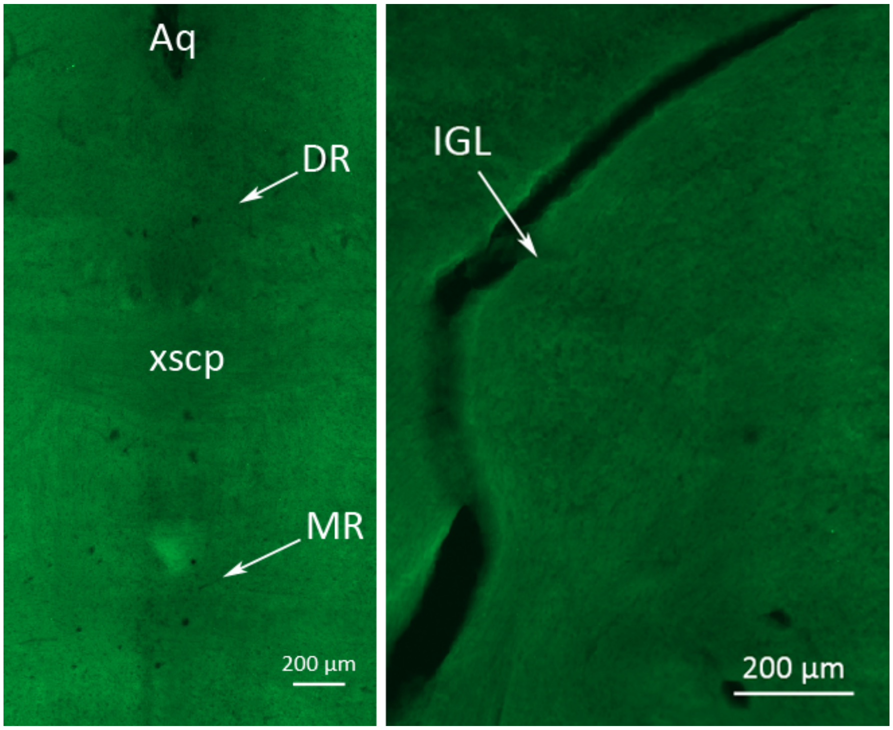

Figure S12. Absence of pTau in other structures that project to the SCN and SPZ in TAPP mice with circadian dysfunction. Sections from the same series in which immunohistochemistry revealed AT8 labeling in the LPB of mice with circadian dysfunction did not show hyperphosphorylated Tau (pTau) in the dorsal and median raphe nuclei (right, DR and MR) of the midbrain or the intergeniculate leaflet (IGL) of the thalamus. Importantly, these structures are well-established inputs to the suprachiasmatic nucleus (SCN) and subparaventricular zone (SPZ) of the circadian system. Aq, cerebral aqueduct. xscp, decussation of the superior cerebellar peduncle. n=5 mice.

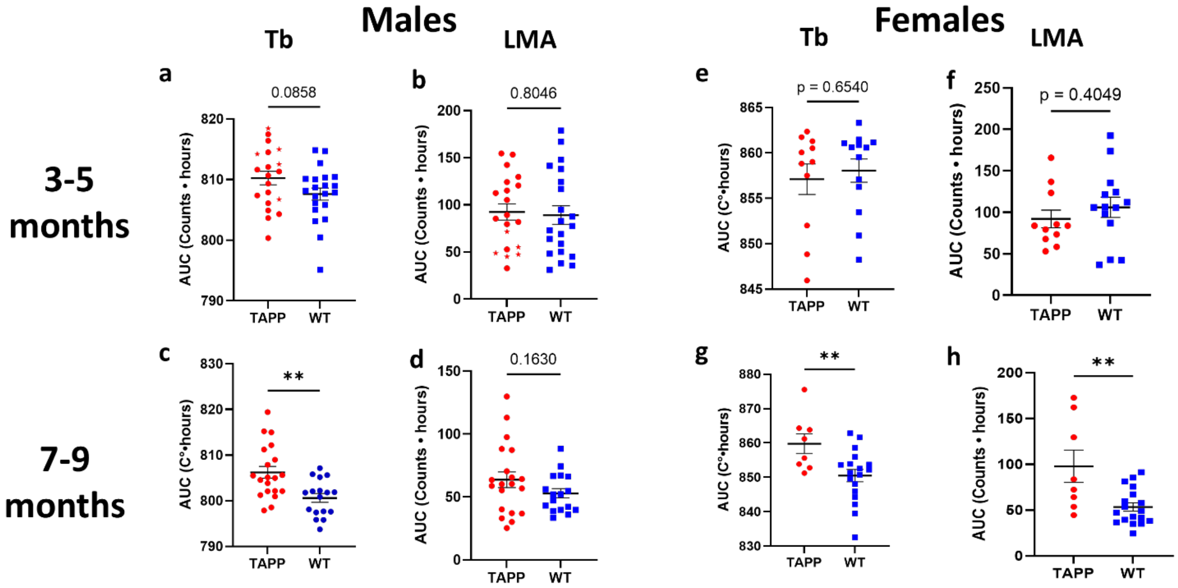

<u>Figure S13</u>. **Male and female 7-9mo, but not 3-5mo, TAPP mice have increased Area under the Curve of Tb and LMA rhythms in a sex dependent manner. a,b)** 3-5mo TAPP males (red circles and stars, n=20 mice) and 3-5mo WT males (blue squares, n=21 mice) have similar area under the curve (AUC) for body temperature (Tb) [Two-tailed unpaired t-test, $t_{(39)}$=1.763] and locomotor activity

(LMA) [Two-tailed unpaired t-test, $t_{(39)}$=0.2491] rhythms. **c,d)** 7-9mo TAPP males (red circles, n=20 mice) have increased AUC for Tb rhythms [Two-tailed unpaired t-test, $t_{(35)}$=3.354, **p=0.0019], but not for LMA rhythms compared to WT mice (blue squares, n=17 mice) [Two-tailed unpaired t-test, $t_{(35)}$=1.1425]. **e,f)** 3-5mo TAPP females (red circles, n=11 mice) and 3-5mo WT females (blue squares, n=13 mice) have similar AUC for Tb [Two-tailed unpaired t-test, $t_{(22)}$=0.4544] and LMA rhythms [Two-tailed unpaired t-test, $t_{(22)}$=0.8485]. **g,h)** 7-9mo TAPP females (red circles, n=8 mice) have increased AUC compared to 7-9mo WT females (blue squares, n=18 mice) for both Tb rhythms [Two-tailed unpaired t-test, $t_{(24)}$=2.810, **p=0.0097] and LMA rhythms [Two-tailed unpaired t-test, $t_{(24)}$=3.305, **p=0.0030].

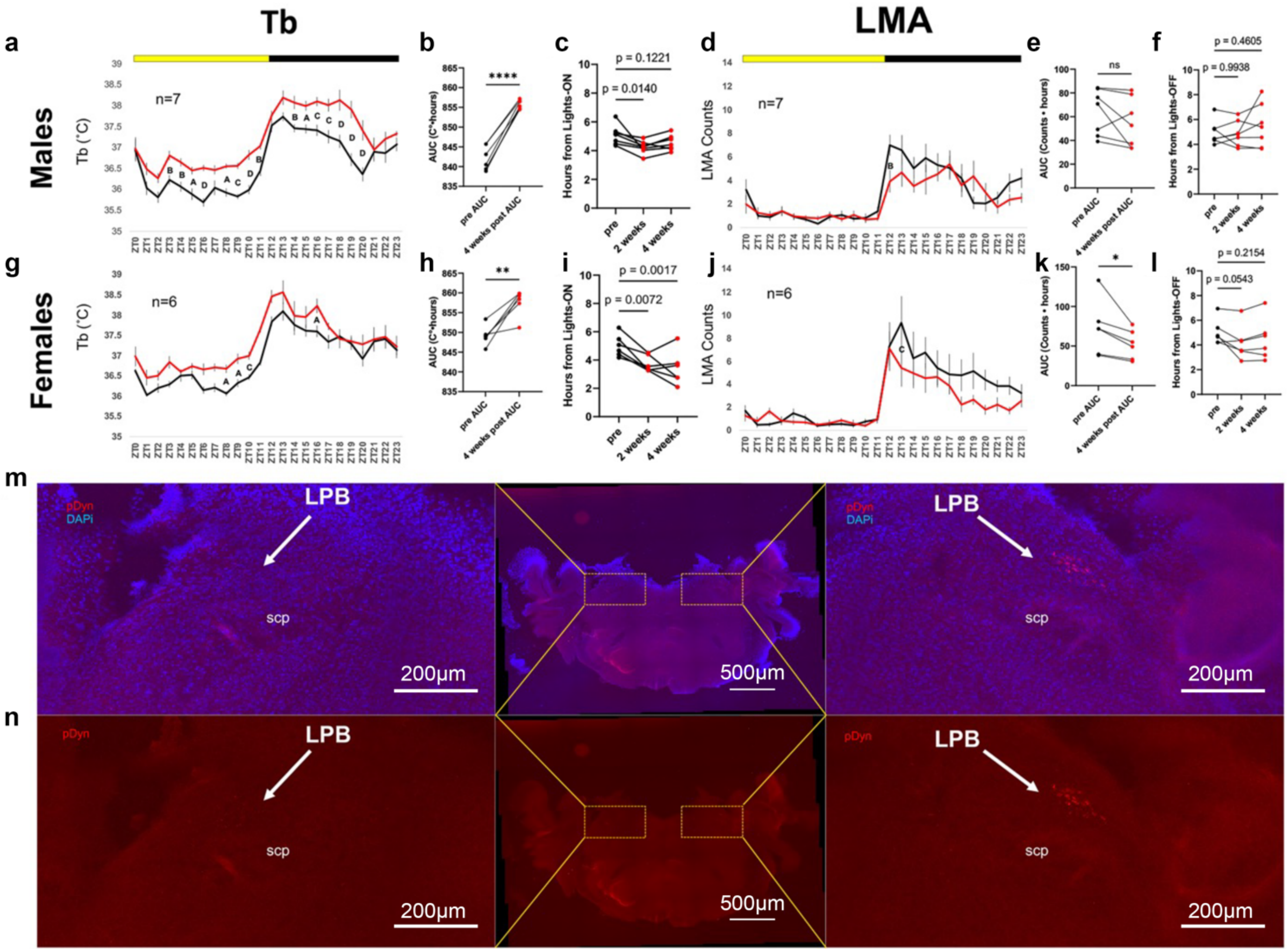

<u>Figure S14.</u> Genetically-targeted LPB^{dyn} ablation persistently increases Tb. **a)** Lateral parabrachial dynorphin (LPB^{dyn})-ablated males exhibited increased body temperature (Tb) throughout the light and dark phases at 4 weeks post-injection (red=post-injection, black=pre-injection) [Two-way RM ANOVA, Interaction: $F_{(23,138)}$=3.239, p<0.0001, Sidak's *post hoc*: ZT3, ^{B}p=0.0076; ZT4, ^{B}p=0.0084; ZT5, ^{A}p=0.0115; ZT6, ^{D}p<0.0001; ZT8, ^{B}p=0.0029; ZT9, ^{C}p=0.0002; ZT10, ^{D}p<0.0001; ZT11, ^{B}p=0.0065; ZT14, ^{B}p=0.0037; ZT15, ^{A}p=0.0149; ZT16, ^{C}p=0.0007; ZT17, ^{C}p=0.0001; ZT18-ZT20, ^{D}p<0.0001]. **b)** Tb area under the curve (AUC, n=7 mice) increased from pre- to post-injection [Two-tailed paired t-test, $t_{(6)}$=15.1, ****p<0.0001]. **c)** LPB^{dyn}-ablated males (n=7 mice) showed earlier Tb acrophases at two but not four weeks post-injection [One-way RM ANOVA, $F_{(2,12)}$=5.406, p=0.0212, Tukey's multiple comparison *post hoc*]. **d)** LPB^{dyn}-ablated males showed LMA differences only at ZT12 four weeks post-injection [Two-way RM ANOVA, Interaction: $F_{(23,138)}$=2.021, p=0.0069, Sidak's *post*

hoc: [B]p=0.0036]. **e)** LPB^{dyn}-ablated males (n=7 mice) showed no difference in LMA AUC [Two-tailed paired t-test, $t_{(6)}$=1.520, p=0.1794]. **f)** LPB^{dyn}-ablated males (n=7 mice) showed no LMA acrophase changes [One-way RM ANOVA, $F_{(2,12)}$=0.9949, p=0.3983, Tukey's multiple comparison *post hocs*]. **g)** LPB^{dyn}-ablated females exhibited increased Tb at multiple timepoints during the light and dark phases (red=post-injection, black=pre-injection) [Two-way RM ANOVA, Interaction: $F_{(23,115)}$=1.759, p=0.0275, Sidak's post hoc: ZT8, [A]p=0.0202; ZT9, [A]p=0.0272; ZT11, [C]p=0.0007; ZT12, [A]p=0.0157]. **h)** LPB^{dyn}-ablated females (n=6 mice) exhibited increased Tb AUC four weeks post-injection [Two-tailed paired t-test, $t_{(5)}$=5.059, **p=0.0039]. **i)** LPB^{dyn}-ablated females (n=6 mice) exhibited earlier Tb bathyphases at two and four weeks post-injection [One-way RM ANOVA, $F_{(2,10)}$=12.42, p=0.0019, Tukey's multiple comparison *post hocs*]. **j)** LPB^{dyn}-ablated females showed LMA changes only at ZT13 [Two-way RM ANOVA, Interaction: $F_{(23,115)}$=2.120, p=0.0050, Sidak's *post hoc*: [C]p=0.0002]. **k)** LPB^{dyn}-ablated females (n=6 mice) exhibited slightly decreased LMA AUC at four weeks post-injection [Two-tailed paired t-test, t(5)=2.745, *p=0.0406]. **l)** LPB^{dyn}-ablated females (n=6 mice) exhibited no LMA acrophase changes [One-way RM ANOVA, $F_{(2,10)}$=3.493, p=0.0707]. **m)** *pDyn* (red) in situ hybridization with DAPi (blue, nuclei) in bilateral LPB (white arrow). **n)** *pDyn* in situ hybridization in bilateral LPB (white arrow). Successful LPB^{dyn} ablation in left LPB, but not in right LPB, verifies reliability of our in situ protocol in revealing absence of *pDyn*. scp: superior cerebellar peduncle. n=13 mice, 7 males and 6 females.
